# Solid State Fermentation—A Promising Approach to Produce Meat Analogues

**DOI:** 10.3390/foods14101820

**Published:** 2025-05-20

**Authors:** Agata Milcarz, Joanna Harasym

**Affiliations:** 1 Department of Biotechnology and Food Analysis, Wroclaw University of Economics and Business, Komandorska 118/120, 53-345 Wroclaw, Poland; agata.milcarz@ue.wroc.pl; 2 Adaptive Food Systems Accelerator-Science Centre, Wroclaw University of Economics and Business, Komandorska 118/120, 53-345 Wroclaw, Poland

**Keywords:** solid-state fermentation, meat analogues, fermentation, plant-based protein

## Abstract

The increasing demand for sustainable dietary options has intensified the development of plant-based meat analogues. Despite growing market availability, these products often fail to replicate conventional meat’s sensory and nutritional properties. Solid-state fermentation (SSF) has emerged as a promising biotechnological approach to enhance the quality of plant-derived protein ingredients. This review summarizes recent findings on the use of SSF in meat analogue production, focusing on microbial strains, substrate selection, and fermentation conditions. The reviewed studies indicate that SSF improves protein digestibility, enhances essential amino acid profiles, reduces anti-nutritional factors, and generates desirable flavour compounds. Furthermore, SSF offers advantages over submerged fermentation in energy and water efficiency, supporting its application in sustainable food processing. The findings highlight SSF’s potential to address key limitations of current meat alternatives and its relevance for developing nutritionally adequate and sensory-appealing products. Integration of SSF into plant-based protein processing may play a critical role in advancing environmentally friendly protein systems.

## 1. Introduction

**Figure 1:**
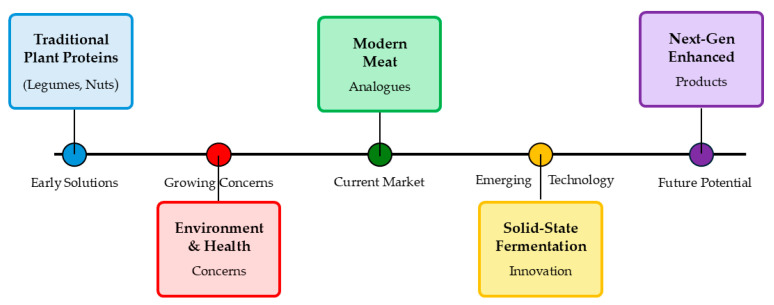
Evolution of plant-based meat alternatives and SSF technology.

The escalating global demand for meat and increasing concerns regarding the environmental and ethical implications of traditional livestock farming have spurred significant interest in alternative protein sources. This review explores the potential of solid-state fermentation as a promising avenue for producing meat analogues, addressing both the sustainability challenges and the nutritional requirements of a growing population. While meat is a vital protein source, it is also associated with unsustainable production practices and potential health risks stemming from excessive consumption, particularly in developed nations. Plant-based meat alternatives have emerged as a viable solution, mitigating adverse effects on human health and the environment compared to conventional meat, mainly processed variants [1]. Meat analogues are not plant-based, protein-rich products; meat analogues are meat-looking/tasting substitutes, with fibrillar characteristics of meat texture and mimicking the colour of meat. The EAT-Lancet Commission advocates a dietary shift towards plant-based proteins like legumes, nuts, and beans to enhance environmental and public health. These alternatives are gaining traction due to the rising global population, limited resources, ethical considerations, and health concerns [2,3]. However, ensuring these alternatives meet consumers’ sensory expectations and nutritional needs remains a key challenge. The evolution of SSF fermentation for meat analogue production is shown in Figure 1.

The production of meat analogues is gaining considerable momentum, with numerous products entering the market that mimic the sensory attributes of animal-based meats [4]. Fermentation technologies, particularly solid-state fermentation, present a unique opportunity to enhance these meat alternatives’ nutritional value, sensory properties, and overall quality [5]. As shown in Table 1, solid-state fermentation offers multiple advantages over conventional processing methods, positioning it as a promising technology for next-generation meat alternatives.

Despite the growing market for plant-based meat alternatives, achieving products that match the sensory properties and nutritional profiles of conventional meat remains challenging. Recent innovations point to integrating fermentation technologies, particularly solid-state fermentation (SSF), as a game-changer in addressing these limitations [6].

SSF allows the utilization of agricultural by-products such as wheat bran, soybean meal, or okara, converting them into high-value protein ingredients through microbial action, often involving filamentous fungi or bacterial cultures [7]. This method supports the development of meat analogues with improved protein bioavailability and digestibility, and is enriched in bioactive compounds, flavour precursors, and desirable textural characteristics [5,6].

SSF enables the bioconversion of complex plant matrices into substrates with reduced anti-nutritional factors, like phytic acid and tannins, while promoting the accumulation of essential amino acids and vitamins [7]. The formation of flavour-active metabolites during fermentation, including short-chain fatty acids and volatile aromatic compounds, mimics the characteristic umami and meaty notes of animal-derived products [4]. This positions SSF as a sustainable and efficient method to improve the sensory appeal of plant-based analogues, meeting consumer expectations for taste, texture, and nutritional adequacy.

**Figure 2:**
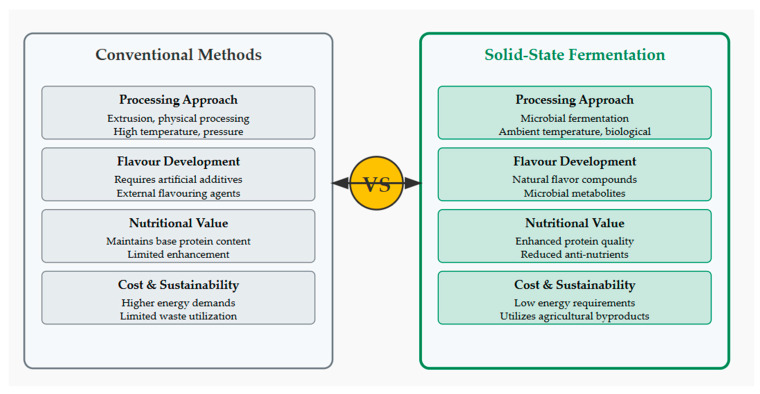
Conventional methods and solid-state fermentation for meat analogue production [5,6,7,9].

The key differences between conventional meat analogue production methods and SSF highlight why SSF represents a transformative approach for the industry, as shown in Figure 2.

While submerged fermentation has been widely explored, SSF is gaining recognition for its lower water and energy demands, aligning with circular economy principles and green biotechnology [9]. The potential to tailor fermentation conditions (such as micro-organism selection, substrate composition, and process parameters) offers vast opportunities to innovate meat analogue development and optimize product formulations [9].

Therefore, this review aims to highlight the role of solid-state fermentation in developing next-generation meat alternatives, emphasizing its technological potential, environmental advantages, and contributions to enhancing nutritional and sensory quality.

## 2. Fundamentals of Solid-State Fermentation

Solid State Fermentation (SSF) is a distinctive bioprocessing technique where micro-organisms grow on solid substrates with minimal free water, unlike Submerged Fermentation (SMF), which is performed in liquid media. SSF creates environments resembling natural habitats where many micro-organisms, particularly filamentous fungi, have evolved [10]. The defining characteristic of SSF is the growth of micro-organisms on water-insoluble substrates with sufficient moisture to support metabolism without exceeding the water-binding capacity of the solid matrix, which creates a three-phase system: solid substrate particles, a thin liquid film, and gas-filled pores for oxygen transfer often yielding higher productivities and more distinct metabolite profiles than SMF [11]. The key differences between SSF and mF are summarized in Table 2.

**Figure 3:**
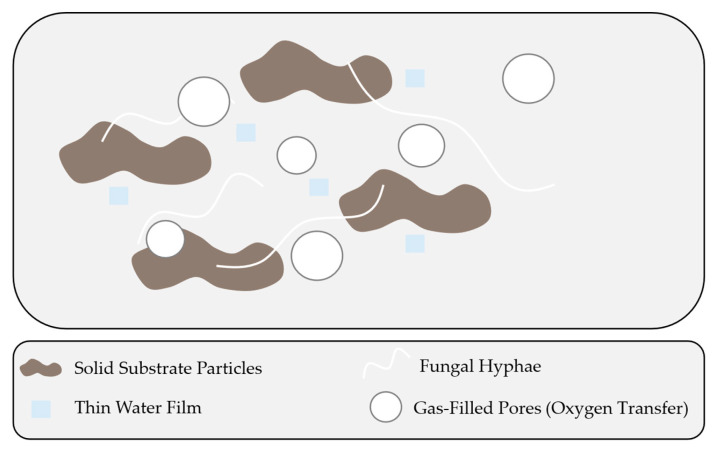
Three-phase structure of solid-state fermentation.

The spatial heterogeneity of SSF (Figure 3) creates diverse microenvironments within a single bioreactor, sometimes supporting microbial succession patterns or enabling stable co-cultivation of multiple micro-organisms.

**Figure 4:**
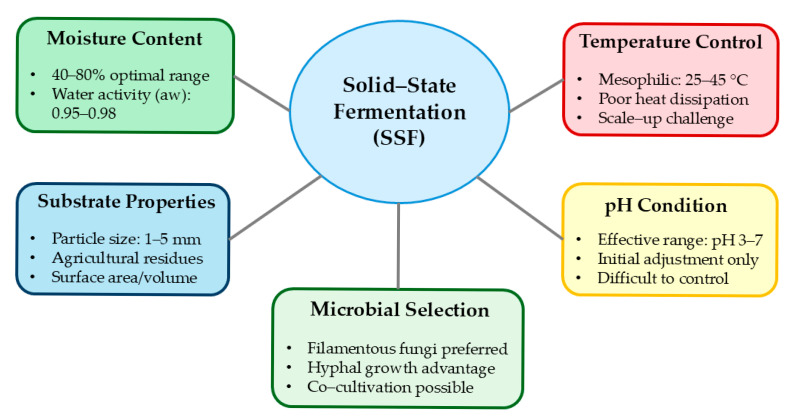
Critical parameters in solid-state fermentation.

Equipment designs for SSF range from simple tray systems to sophisticated bioreactors. Scale-up represents perhaps the most significant challenge limiting wider industrial adoption of SSF [12]. Unlike SmF, where scale-up follows well-established engineering principles, SSF scale-up is complicated by the heterogeneous nature of the process and difficulties in heat removal from large substrate beds. The critical parameters that need to be controlled during SFF are shown in Figure 4.

Despite these challenges, advances in bioreactor design, process monitoring, and control strategies have significantly improved the industrial viability of SSF processes, positioning them as a viable alternative to SmF for applications including protein-rich biomass production suitable for meat analogue development.

**Table 2:** Comparison of solid-state fermentation (SSF) vs Submerged Fermentation (SmF).

Parameter	Solid-State Fermentation (SSF)	Submerged Fermentation (SmF)	**Refs.**
Medium Composition	Solid substrates with minimal free water	Liquid media with dissolved nutrients	[10,11]
Water Content	Low (40–80% moisture)	High (>95% water)	[13,14]
Oxygen Transfer	Through gas-filled pores in substrate matrix	Via mechanical agitation and aeration	[11]
Preferred Micro-organisms	Filamentous fungi (hyphal growth mode)	Bacteria, yeasts, unicellular organisms	[15]
Growth Kinetics	Often linear growth patterns	Exponential growth patterns	[16]
Heat Transfer	Poor (low thermal conductivity)	Efficient (convection in liquid)	[17]
pH Control	Difficult (relies on initial adjustment)	Relatively easy (continuous monitoring)	[10]
Scale-up Complexity	High (heterogeneous environment)	Moderate (well-established principles)	[18]
Capital Costs	Lower	Higher	[16]
Product Yield/Concentration	Often higher for certain processes	Variable, depending on application	[12]
Common Applications	Enzymes, bioactive compounds, biomass	Antibiotics, organic acids, recombinant proteins	[10,12]

## 3. Substrates for SSF in Meat Analogue Production

The selection of appropriate substrates is critical for solid-state fermentation (SSF) systems designed for meat analogue production. Unlike traditional SSF applications primarily focusing on enzyme or metabolite production, meat analogue development requires substrates supporting microbial growth while providing substantial protein content, appropriate texture-forming capabilities, and desirable sensory attributes [13].

Cereal grains and legumes constitute the primary substrate classes employed in SSF for meat analogues, with legumes being particularly valuable due to their naturally high protein content. Soybeans and soybean derivatives dominate commercial applications, building upon their long history in traditional fermented foods like tempeh and their established role in conventional meat alternative products [14]. The protein content of soybeans, typically 35–40% on a dry weight basis, provides an excellent starting point for developing nutritionally adequate meat substitutes. Beyond protein quantity, soybeans offer a relatively complete amino acid profile compared to other plant proteins, though they remain somewhat limiting in sulphur-containing amino acids like methionine [18].

Other legumes, including chickpeas, lentils, fava beans, and lupins, have garnered increasing attention as alternative substrates. Chickpeas (20–22% protein) have demonstrated excellent fermentability in SSF systems while contributing unique flavour profiles [17]. Cereal grains, while generally having lower protein content than legumes, serve essential functional roles in SSF substrate formulations. Wheat, with approximately 12–14% protein content, contributes vital glutenin and gliadin proteins that impart viscoelastic properties crucial for developing fibrous, meat-like textures [15]. Despite its relatively low protein content (7–9%), rice offers excellent fermentability and neutral flavour profiles that serve as effective matrices for incorporating flavour compounds during fermentation [13] (Table 3).

Agricultural by-products and waste streams present compelling alternative substrate options, aligning sustainability objectives with functionality requirements. Cereal brans, particularly wheat bran, provide excellent structural support for fungal growth in SSF while contributing significant fibre content and micronutrients to the final product [16]. Recent studies have highlighted the growing interest in utilizing mixed substrate systems combining legume flours with agricultural by-products to enhance the protein density and functional properties of the SSF matrix. For instance, the co-fermentation of soybean flour and wheat bran has improved microbial growth and final product texture due to synergistic nutrient availability and enhanced aeration properties [2]. Such hybrid substrates also enable the tailoring of fermentation conditions to support specific microbial consortia, offering new avenues for designing products with optimized flavour and nutritional profiles. Oilseed cakes and meals, residual materials from oil extraction processes, offer protein contents sometimes exceeding 45%, along with residual lipids that can enhance the sensory properties of meat analogues [19].

Various pre-treatment methods have been developed to improve substrate accessibility in SSF systems. Physical pre-treatments, including milling, grinding, and extrusion, increase surface area and disrupt cell wall structures, thereby enhancing the accessibility of intracellular nutrients to microbial action [16]. Particle size optimization represents a particularly critical consideration; Rahardjo et al. [20] demonstrated that soybean particles between 2 and 4 mm in diameter provided an optimal balance between surface accessibility and maintenance of inter-particle void spaces necessary for oxygen diffusion during tempeh production.

Enzymatic pre-treatments represent more targeted approaches to enhancing substrate accessibility. The application of cellulases and hemicellulases facilitates partial hydrolysis of plant cell wall components, releasing trapped nutrients and creating pathways for hyphal penetration in filamentous fungi-based processes [21].

Emerging bioprocessing strategies also explore the use of underutilized crops and pseudocereals such as quinoa, amaranth, and buckwheat in SSF systems aimed at meat analogue production. These substrates offer unique amino acid profiles, including higher levels of lysine than most cereals, and their compatibility with filamentous fungi like *Rhizopus* and *Monascus* opens possibilities for functional food innovation [22]. Integrating such novel raw materials into SSF-based meat analogues may contribute to nutritional diversification and resilience in protein supply chains.

## 4. Microorganisms in SSF for Meat Analogues

Microorganism selection is critical for solid-state fermentation (SSF) in meat analogue production, requiring efficient conversion of plant substrates into protein-rich biomass with desirable sensory and nutritional properties [23] (Table 4).

Filamentous fungi dominate commercial SSF applications due to their hyphal growth mode, which effectively colonizes solid substrates. *Rhizopus* species (*R. oligosporus, R. oryzae*), extensively used in tempeh production, exhibit rapid growth and secrete amylolytic and proteolytic enzymes while synthesizing B-vitamins [24,25]. Their mycelial networks create meat-like textures through physical hyphal entanglement with substrate particles, yielding coherent, sliceable products with desirable mouthfeel [25].

*Aspergillus oryzae* produces diverse hydrolytic enzymes (amylases, proteases, lipases) that enhance substrate digestibility and flavour development [26]. Unlike surface-growing *Rhizopus*, *Aspergillus* penetrates deeper into substrate particles, extensively modifying internal structures—valuable for recalcitrant substrates like cereal brans or lignocellulosic materials [13,26].

*Neurospora intermedia*, used in Indonesian *oncom* production, grows rapidly on challenging substrates including peanut press cake, coconut residue, and cassava by-products [27]. Its carotenoid-derived orange pigmentation offers potential for developing visually appealing red meat analogues [27].

**Table 4:** Micro-organisms and their roles in SSF-based meat analogues production.

Category	Examples	Key Functionalities	Refs.
Filamentous Fungi	*Rhizopus* (*R. oligosporus*, *R. oryzae*)	Rapid growth, enzyme secretion (amylases, proteases), B-vitamin synthesis, meat-like texture formation	[24,25]
	*Aspergillus* (*A. oryzae*)	Hydrolytic enzyme production (amylases, proteases, lipases), deep substrate penetration, enhanced digestibility	[28]
	*Neurospora* (*N. intermedia*)	Fast growth on diverse substrates, carotenoid production (natural pigmentation)	[29]
Bacteria	*Bacillus* (*B. subtilis*)	Protease production, protein hydrolysis, flavour enhancement, improved digestibility	[30]
	Lactic Acid Bacteria (*Lactobacillus*, *Pediococcus*, *Lactococcus*)	Lactic acid production, pH reduction, microbial inhibition, preservation	[31]
Mixed Cultures	Co-cultures (e.g., *Rhizopus* + LAB)	Combination of complementary metabolic pathways for enhanced texture, flavour, and safety	[32]

While filamentous fungi dominate current applications, several bacterial species demonstrate promising capabilities for meat analogue production via SSF. *Bacillus subtilis* and related species feature prominently in traditional fermented foods like natto and contribute valuable functionalities in meat analogue contexts [30]. These organisms produce extracellular proteases that extensively hydrolyse substrate proteins, releasing free amino acids and peptides that enhance flavour profiles while improving protein digestibility.

Lactic acid bacteria (LAB), including *Lactobacillus*, *Pediococcus*, and *Lactococcus* species, typically play secondary roles in SSF systems for meat analogues but contribute valuable functionalities. These organisms rapidly acidify the substrate through lactic acid production, inhibiting the growth of undesirable microorganisms while contributing to preservation and safety [31].

Mixed culture fermentations represent a promising frontier in developing next-generation meat analogues via SSF, potentially combining complementary metabolic capabilities of multiple microorganisms. Traditional fermented foods like tempeh and *oncom* often involve complex microbial consortia rather than pure cultures, suggesting unexplored potential in designed co-cultures [32].

The selection criteria for starter cultures in meat analogue SSF must balance multiple considerations. Growth rate represents a primary criterion, with rapid colonisation abilities necessary to outcompete potential contaminating microorganisms while achieving reasonable production timelines. Protein productivity, safety considerations, and sensory contributions significantly influence starter culture selection, particularly on strains that produce desirable flavour compounds, textures, and visual attributes reminiscent of conventional meat products [14].

## 5. Technological Aspects of Meat Analogue Production via SSF

**Figure 5:**
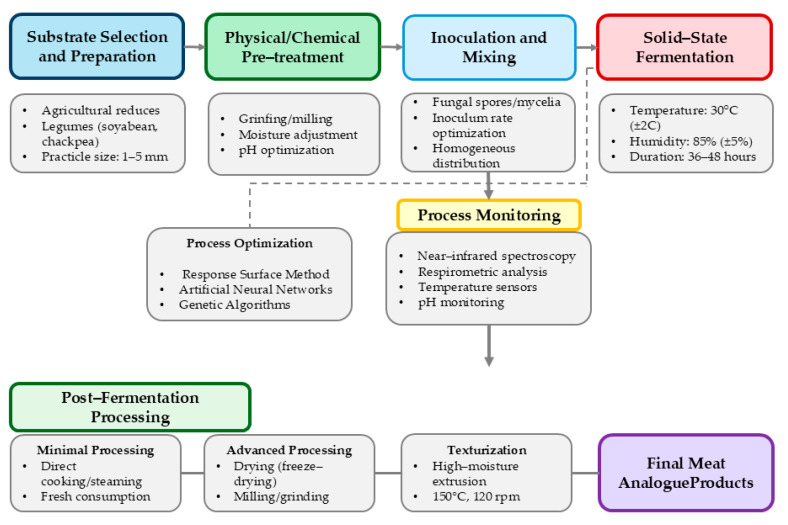
Process flow for SSF-based meat analogue production [19,24,33].

Translating solid-state fermentation (SSF) from laboratory-scale investigations to industrial meat analogue production necessitates careful consideration of numerous technological aspects. Unlike conventional meat alternative manufacturing, which typically relies on established extrusion technologies, SSF-based approaches introduce unique processing considerations related to substrate preparation, fermentation control, post-fermentation processing, and integration with complementary technologies [28] (Figure 5).

SSF process optimization for meat analogues requires multidimensional approaches, primarily focusing on substrate formulation and fermentation parameters. Response surface methodology (RSM) effectively identifies optimal variable combinations in complex systems. Handoyo and Morita [24] optimized tempeh production using RSM, determining optimal conditions (30 °C, 85% RH, 36 h fermentation) that maximized protein content and textural properties. Advanced computational methods, including artificial neural networks (ANNS) and genetic algorithms, offer improved optimization for nonlinear SSF processes with complex variable interactions. Hölker and Lenz [33] demonstrated ANN superiority over RSM in predicting glucoamylase production during *Aspergillus niger* SSF, achieving higher predictive accuracy (Table 5).

However, the practical application of these optimization methods often faces several limitations. The complexity and variability of SSF systems, combined with the high heterogeneity of solid substrates, pose challenges in process reproducibility, monitoring, and control. Scalability remains a major bottleneck, as laboratory-optimized conditions do not always translate directly to pilot or industrial scales [35].

SSF monitoring presents unique challenges due to substrate heterogeneity and sampling difficulties. Near-infrared spectroscopy (NIRS) enables non-invasive monitoring of moisture, protein, and substrate consumption, while respirometric techniques, measuring O_2_ consumption and CO_2_ evolution, indirectly measure microbial activity correlating with growth and metabolism [10].

Despite these advances, real-time control and standardization of SSF processes remain difficult. In homogeneous microbial growth, variable moisture gradients, and limited heat and mass transfer can lead to inconsistent product quality, limiting broader adoption of SSF in large-scale applications [23].

**Figure 6:**
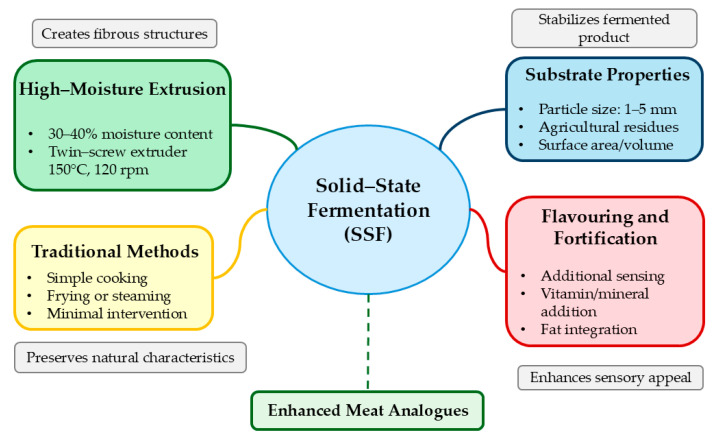
Integration of SSF with complementary technologies.

Post-fermentation processing converts fermented substrates into consumer products. Traditional tempeh requires minimal processing—simple cooking before consumption [25]. Shelf-stable products need additional steps, including controlled drying, with freeze-drying preserving protein functionality and flavour compounds. Texturization enhances the meat-like properties of SSF products. High-moisture extrusion of fermented substrates creates fibrous structures resembling muscle tissue [19]. Extrusion of fermented soybean (30–40% moisture, 150 °C, 120 rpm) produces aligned protein fibres mimicking chicken breast texture. Integrating SSF with complementary technologies advances meat analogue development. Combining SSF with high-moisture extrusion synergistically leverages fermentation-derived flavours and extrusion-based texturization [34]. Fermentation-induced partial protein hydrolysis facilitates realignment during extrusion while preserving thermally stable flavour compounds (Figure 6).

Emerging additive manufacturing technologies, mainly 3D food printing, offer intriguing possibilities for creating structurally complex meat analogues incorporating fermented components. Combining 3D printing’s structural control with fermentation’s flavour development capabilities presents up-and-coming opportunities for replicating complex meat products like marbled steaks that remain challenging for conventional technologies.

Quality control and standardization approaches represent essential considerations for industrial-scale production of SSF meat analogues. Hazard Analysis Critical Control Points (HACCP) frameworks adapted specifically for fungal fermentations identify critical control points, including initial substrate contamination levels, inoculum quality, temperature control during fermentation, and moisture management [24].

Nonetheless, industrial implementation requires overcoming technical, economic, and regulatory barriers. A more robust understanding of process dynamics, as well as standardized protocols, will be crucial to support commercial scalability [35].

## 6. Nutritional and Functional Properties of SSF-Derived Meat Analogues

The nutritional and functional properties of solid-state fermentation (SSF) derived meat analogues represent critical determinants of their consumer acceptance, market positioning, and potential health impacts. Unlike conventional meat alternatives primarily developed through the physical processing of plant proteins, SSF products undergo complex biochemical transformations that significantly modify their nutritional profiles, bioactive compound content, and techno-functional characteristics (Table 6) [32].

Due to multiple complementary mechanisms, protein quality in SSF-derived meat analogues typically exceeds that of their unfermented counterparts. The microbial biomass contributes high-quality protein with amino acid compositions often complementary to plant protein limitations [25]. Analysis of *Rhizopus oligosporus* biomass reveals exceptionally high levels of lysine and methionine, amino acids frequently limiting plant proteins, potentially enhancing the biological value of the composite protein in fermented products [15].

SSF provides significant nutritional enhancements to meat analogues. Sparringa and Owens [36] showed fungal biomass contributes 12–15% of total protein, especially essential amino acids. Protein digestibility improves by 15–25% in legume substrates through antinutritional factor degradation (phytates, tannins), enzymatic hydrolysis, protease inhibitor inactivation, and protein structure modification [37,38]. Micronutrient bioavailability increases substantially during SSF. Eklund-Jonsson et al. [39] demonstrated *R. oligosporus* fermentation reduced barley phytate by 97%, enhancing iron, zinc, and calcium accessibility. B-vitamin synthesis also occurs, with riboflavin increasing 2.5-fold and niacin 2-4-fold during *Rhizopus* fermentation [40].

Beyond basic nutrition, SSF generates bioactive compounds, including phenolic compounds and peptides with antioxidants, antidiabetic, anticancer, anti-inflammatory, and ACE-inhibitory activities [38]. SSF-derived products often demonstrate superior sensory characteristics while offering comparable or enhanced nutritional profiles to other alternative proteins like conventionally processed plants or single-cell proteins. The natural flavour development during fermentation provides distinctive savoury notes that are difficult to achieve through conventional processing or flavour addition alone. Additionally, the fibrous textures created through mycelial network formation offer structural attributes that complement or potentially exceed those achievable through conventional extrusion or spinning technologies [14].

## 7. Sensory Attributes and Consumer Acceptance

The sensory characteristics of solid-state fermentation (SSF) derived meat analogues represent critical determinants of consumer acceptance and market success. Unlike conventional meat alternatives that rely on exogenous flavour additives and extensive processing to achieve meat-like properties, SSF products develop complex sensory attributes through natural microbial metabolic activities [26] (Table 7). These natural transformations can yield unique and desirable sensory properties, though they simultaneously present challenges related to standardization and control. Recent studies have highlighted SSF’s potential to naturally generate desirable flavour and texture characteristics without requiring additional chemical additives or texturizing agents [2].

**Figure 7:**
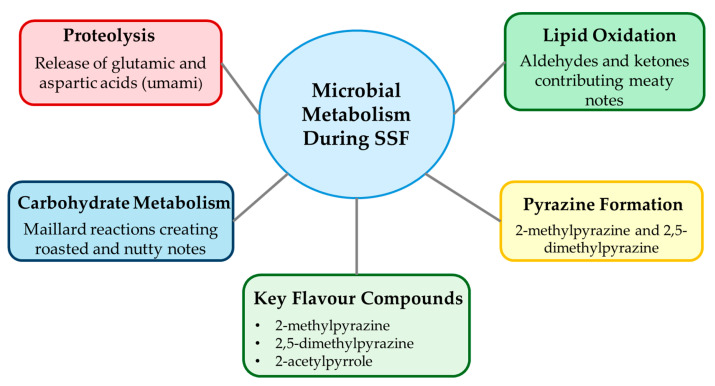
Flavour development pathways in SSF-derived meat analogues.

Flavour development during SSF occurs through multiple complementary biochemical pathways that generate complex taste and aroma profiles (Figure 7).

Proteolytic activity releases amino acids contributing to taste, with glutamic and aspartic acids providing umami notes essential for meat-like flavour [41]. Feng et al. [42] identified over 45 volatile compounds in Rhizopus-fermented barley, including 2-methylpyrazine, 2,5-dimethylpyrazine, and 2-acetylpyrrole, which contribute roasted, nutty, and meat-like aromas.

Volatile profiles are strain-dependent and modifiable through inoculum selection and fermentation conditions [6]. Co-culture approaches combining Rhizopus with yeasts or lactic acid bacteria enhance flavour complexity and mask undesirable plant matrix off-notes [43].

**Figure 8:**
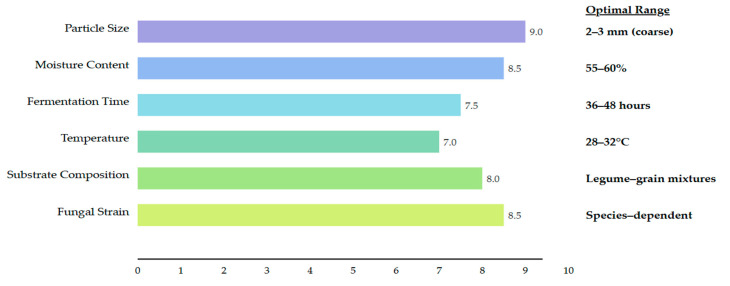
Factors influencing texture development in SSF-derived meat analogues.

The texture characteristics of SSF products derive primarily from fungal mycelia networks that penetrate and bind substrate particles, creating natural fibrous structures resembling meat without extensive extrusion or spinning processes [19]. Mycelial network density, controlled by fermentation time, temperature, and substrate composition, determines firmness, cohesiveness, and chewiness. Manipulating fermentation variables, including substrate porosity and particle distribution, enables texture customization for specific consumer preferences (Figure 8) [6].

Substrate particle size significantly influences SSF texture, with coarser particles (2–3 mm) yielding firmer, meat-like textures than finer particles (<1 mm) that produce softer products [24]. Moisture content also affects texture—higher levels (65–70%) promote extensive mycelial growth but may reduce structural integrity compared to moderate moisture (55–60%). Colour presents challenges as SSF typically produces off-white to greyish products, contrasting with meat’s red/pink/brown hues [25]. Natural pigmentation strategies using *Monascus purpureus* and *Neurospora* strains offer clean-label solutions [2]. *N. intermedia* produces orange-red carotenoid pigments suitable for salmon/tuna analogues, while *M. purpureus* generates red pigments resembling beef [2].

**Figure 9:**
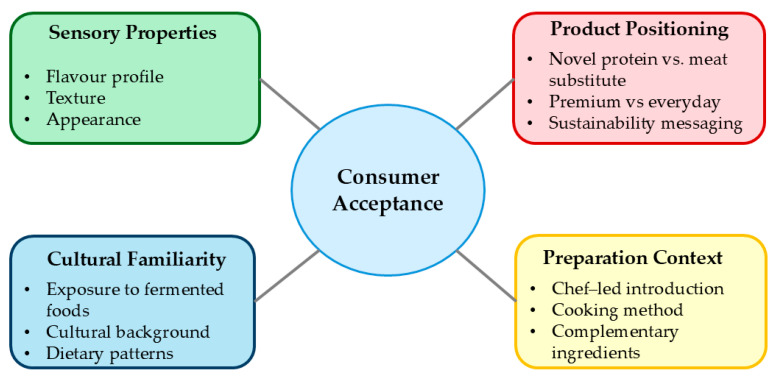
Factors influencing consumer acceptance of SSF-derived meat analogues [27,33,34,44,45].

Consumer perception studies reveal complex relationships between sensory properties, familiarity, and acceptance of SSF-derived meat analogues (Figure 9).

Consumer acceptance of SSF-based meat analogues correlates with familiarity with fermented foods, with traditional fermented food consumers showing higher acceptance rates. Product positioning affects the perception SSF products marketed as novel proteins receive higher acceptance than those positioned as meat substitutes, especially among meat consumers. Chef-led presentations enhance associations with premium, natural, and sustainable qualities [43]. Sensory improvement strategies include substrate blending, controlled pre-fermentation, and post-fermentation enhancements (marination, smoking). Precision fermentation and metabolomic monitoring increase reproducibility and enable precise sensory profile customization [6].

Heat treatment plays a crucial role in determining the final sensory quality of SSF-derived meat analogues. Cooking processes such as steaming, baking, or frying can enhance the release of volatile compounds developed during fermentation, intensifying umami and roasted flavours. Moreover, heat application modifies the texture—typically increasing chewiness and cohesiveness—making the final product more meat-like and palatable for consumers [36,46]. Thus, evaluation of sensory characteristics post-cooking is essential for assessing the real-world eating quality and consumer acceptance of SSF products.

## 8. Food Safety Considerations

Food safety represents a critical dimension of solid-state fermentation (SSF) meat analogue production that requires systematic attention throughout product development and manufacturing processes (Table 8).

Unlike conventional extrusion-based alternatives, SSF products require unique safety considerations, including starter culture purity, contaminant management, mycotoxin prevention, and allergenicity control [46]. Microbial safety requires validated starter cultures, with commercial production favouring defined cultures over traditional undefined mixed cultures [47]. Organisms should possess GRAS status or a documented safe use history. Feng et al. [42] showed commercial lyophilized *R. oligosporus* offered superior safety and consistency compared to traditional hibiscus leaf-derived starters [1].

**Figure 10:**
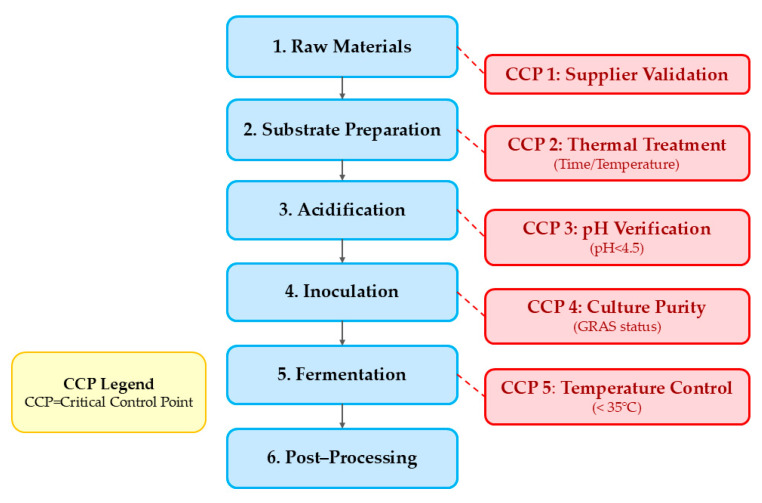
HACCP framework for SSF-derived meat analogue production [44].

Substrate preparation represents a critical safety control. Near-neutral pH of SSF substrates necessitates thermal treatments (boiling, steaming, autoclaving) to reduce microbial loads and inactivate antinutritional factors [1,25]. Nout et al. [48] demonstrated pre-fermentation acidification to pH 4.5 effectively inhibited pathogens while allowing normal Rhizopus development, establishing critical safety hurdles during early fermentation. Implementing HACCP (Hazard Analysis Critical Control Points) principles provides a framework for addressing safety concerns of SSF meat analogue production (Figure 10).

Steinkraus [30] identified critical control points specific to tempeh production, including substrate thermal treatment, acidification (pH < 4.5 before fungal inoculation), and fermentation temperature control (maintaining < 35 °C to prevent growth of thermophilic pathogens while supporting desired fungal development).

Mycotoxin risks require particular attention in fungal SSF systems due to the potential for some filamentous fungi to produce these secondary metabolites under certain conditions [46,48].

Fortunately, *Rhizopus* species commonly employed in tempeh and similar fermentations are not known to produce significant mycotoxins, confirming the absence of aflatoxin, ochratoxin, and other common mycotoxins in properly produced tempeh [37]

*Aspergillus* species employed in specific SSF applications present more complex considerations, as some strains within this genus can produce mycotoxins under certain conditions. Therefore, selecting strains with low mycotoxin-producing potential is crucial for ensuring the safety of SSF meat analogues. Industrial strains of *Aspergillus oryzae* employed in koji production have been extensively evaluated and generally lack the genetic capacity for producing aflatoxins or other concerning mycotoxins [38].

Allergenicity considerations for SSF-derived meat analogues encompass both substrate-related and fermentation-induced dimensions. Many common SSF substrates, particularly soybeans and other legumes, contain known allergens that persist through fermentation. Beyond substrate allergens, fungal biomass presents potential allergenic concerns, although thermal processing significantly reduces the allergenic potential of these proteins. Recent studies have provided insights into managing allergenicity in SSF products, highlighting the need to carefully select substrates and processing conditions [1].

Regulatory aspects and compliance frameworks for SSF-derived meat analogues vary significantly between jurisdictions, presenting challenges for international commercialization. Standardized safety validation protocols tailored to SSF-derived meat analogues would facilitate substantial regulatory compliance and market access. Recent reviews have called for harmonized regulatory approaches to support the global trade of SSF meat analogues [39].

## 9. Conclusions

Solid-state fermentation offers a promising and sustainable strategy to enhance plant-based meat analogues’ nutritional and sensory properties. It improves protein quality, reduces anti-nutritional factors, and contributes to flavour development, supporting the creation of clean-label products. However, challenges such as process scalability, substrate consistency, and optimal microbial selection remain. Further research should address these limitations by focusing on fermentation optimization, strain improvement, and process standardization to enable industrial implementation. Incorporating SSF into plant-based protein processing could significantly advance the development of high-quality, functional, and commercially viable meat alternatives, opening new opportunities for innovation in the food industry.

## Figures and Tables

**Table 1:** Key benefits of solid-state fermentation in meat analogue production.

Area	Specific Advantages	Impact on Meat Analogues	Refs.
Nutritional Enhancement	Increased protein content Improved amino acid profile Enhanced digestibility	Better nutritional match to conventional meat	[5,6]
Sensory Properties	Natural flavour development Desirable texture formation Meat-like umami notes	Enhanced consumer acceptance and palatability	[4,5]
Sustainability	Utilizes agricultural byproducts Lower water usage than submerged fermentation Energy-efficient process	Reduced environmental footprint	[7,8]
Functional Properties	Anti-nutritional factor reduction Bioactive compound production Natural preservation effects	Improved health benefits and shelf stability	[6,8]
Economic Benefits	Cost-effective production Waste stream valorization Minimal processing requirements	Lower production costs compared to alternatives	[6,7]

**Table 3:** Substrates and their functional roles in SSF for meat analogues.

Substrate Class	Examples	Protein Content (% d.w.)	Key Properties	Ref.
Legumes	Soybeans, chickpeas, lentils, fava beans, lupins	20–45%	High protein content, rich amino acid profile, good fermentability	[17]
Cereal Grains	Wheat, rice	7–14%	Structural properties (glutenin & gliadin in wheat), neutral flavour profile (rice)	[13]
Agricultural by-products	Wheat bran	Variable	Structural support for fungal growth, high fibre and micronutrient content	[16]
Oilseed Cakes and Meals	Residues from oil extraction (soybean, rapeseed meals)	>45%	High protein content, residual lipids enhancing sensory properties	[19]

**Table 5:** Optimization methods for SSF-based meat analogue production.

Optimization Method	Key Characteristics	Application to SSF	Advantages	Limitations	Applicable Conditions and Practical Limitations	Ref.
Response Surface Methodology (RSM)	Statistical technique that explores relationships between variables and responses	Optimizing temperature (30 °C), relative humidity (85%), and fermentation time (36 h) for tempeh production	Provides visual representation of optimal conditions; identifies interactions between variables	Limited to relatively simple systems with few variables	Best for small-scale studies with few variables; less suited for complex systems	[24]
Artificial Neural Networks (ANNs)	A machine learning approach that models complex nonlinear relationships	Prediction of glucoamylase production in *Aspergillus niger* SSF	Superior predictive accuracy for complex systems; handles highly nonlinear responses	Requires substantial data for training; “black box” nature limits interpretability	Ideal with large datasets; “black-box” may limit insight	[33]
Genetic Algorithms	The evolutionary computational approach that mimics natural selection	Optimizing multi-variable SSF processes with complex interactions	Can search large solution spaces efficiently; not limited by mathematical constraints	Computationally intensive; may converge to local optima	Best for complex, multidimensional problems	[34]
Design of Experiments (DoE)	Structured approach to determine cause-and-effect relationships	Identifying critical process parameters in SSF systems	Reduces experimental burden; systematic approach	May oversimplify complex biological systems	Good for early-phase or screening studies	[13]
Process Analytical Technology (PAT)	Framework for designing, analyzing, and controlling manufacturing	Real-time monitoring of moisture, protein concentration, and substrate consumption	Enables real-time process adjustments; improves consistency	Implementation challenges in heterogeneous SSF systems	Suited for well-equipped setups; costly to implement	[10]
Hybrid Approaches	Combination of multiple optimization techniques	Integration of empirical models with machine learning for comprehensive process optimization	Leverages strengths of multiple methods; improved robustness	Increased complexity; requires multidisciplinary expertise	Great for advanced settings with computational tools	[19]

**Table 6:** Nutritional enhancements in SSF-derived meat analogues.

Nutritional Parameter	Enhancement During SSF	Key Findings from Research	Refs.
Protein Quality	Addition of complementary amino acids	*Rhizopus oligosporus* biomass contributes high levels of lysine and methionine	[20]
Protein Contribution	Increased protein content	Fungal biomass contributes 12–15% of total protein content	[36]
Protein Digestibility	15–25% improvement	Enzymatic hydrolysis, inactivation of protease inhibitors, structural modifications	[37,38]
Mineral Bioavailability	Significant increase	97% reduction in phytate content, increased iron, zinc, and calcium accessibility	[39]
Vitamin Content	2–4-fold increases	2.5-fold increase in riboflavin, 2–4-fold increase in niacin	[40]
Antioxidant Capacity	2–3-fold increases	Enhanced DPPH radical scavenging activity in *Aspergillus oryzae* fermented soybeans	[38]

**Table 7:** Key sensory attributes of SSF-derived meat analogues.

Sensory Attribute	Observations	Influencing Factors	Refs.
Flavour Development	Complex profiles with umami notes; over 45 distinct aroma compounds including 2-methylpyrazine, 2,5-dimethylpyrazine, and 2-acetylpyrrole	Microbial strain selection, fermentation duration, proteolytic activities releasing amino acids	[41,42]
Texture Formation	Natural fibrous structure from mycelial network; meat-like properties without extensive extrusion	Substrate particle size (2–3 mm optimal), moisture content (55–70%), fermentation time and temperature	[19,24]
Colour and Appearance	Typically off-white to greyish appearance; *Neurospora* strains produce orange-red pigments; *Monascus purpureus* generates red pigments	Fungal strain selection, fermentation conditions, substrate composition	[2,25]
Consumer Perception	Higher acceptance when positioned as novel protein sources rather than meat substitutes; cultural background influences acceptance	Familiarity with fermented foods, product positioning, chef-led introduction	[43]
Enhancement Strategies	Substrate blending, co-culture fermentation, post-fermentation treatments (marination, smoking)	Integration of precision fermentation and metabolomic monitoring	[6,43]

**Table 8:** Key food safety considerations in SSF-derived meat analogues.

Safety Consideration	Control Measures	Observations	Refs.
Starter Culture Safety	Use of defined GRAS cultures; validation of purity	Commercial lyophilized cultures show superior consistency and safety compared to traditional starters	[42,47]
Substrate Preparation	Thermal treatments (boiling, steaming, autoclaving); acidification to pH 4.5	Pre-fermentation acidification effectively inhibits pathogen growth while allowing normal Rhizopus development	[25,48]
Process Control	HACCP implementation; temperature control (<35 °C); monitoring of fermentation parameters	Critical control points include substrate thermal treatment, acidification, and fermentation temperature	[44]
Mycotoxin Prevention	Selection of non-mycotoxigenic strains; control of fermentation conditions	Rhizopus species do not produce significant mycotoxins; industrial A. oryzae strains lack genetic capacity for aflatoxin production.	[26,37]
Allergenicity Management	Careful substrate selection; appropriate thermal processing	Thermal processing reduces allergenic potential of fungal biomass proteins	[1]
Regulatory Compliance	Adherence to regional requirements; safety validation protocols	Harmonized regulatory approaches are needed to support global trade of SSF meat analogues	[39]

## Data Availability

No new data were created or analyzed in this study. Data sharing is not applicable to this article.
